# Natural hosts and animal models for Rift Valley fever phlebovirus

**DOI:** 10.3389/fvets.2023.1258172

**Published:** 2023-10-19

**Authors:** Yuqing Xu, Xiao Wang, Lu Jiang, Yixuan Zhou, Yihan Liu, Fei Wang, Leiliang Zhang

**Affiliations:** ^1^Department of Clinical Laboratory Medicine, The First Affiliated Hospital of Shandong First Medical University and Shandong Provincial Qianfoshan Hospital, Jinan, China; ^2^Medical Science and Technology Innovation Center, Shandong First Medical University, Shandong Academy of Medical Sciences, Jinan, China; ^3^Department of Pathogen Biology, School of Clinical and Basic Medical Sciences, Shandong First Medical University and Shandong Academy of Medical Sciences, Jinan, China; ^4^School of Laboratory Animal and Shandong Laboratory Animal Center, Shandong First Medical University and Shandong Academy of Medical Sciences, Jinan, China

**Keywords:** RVFV, RVF, zoonotic, natural host, animal model

## Abstract

Rift Valley fever phlebovirus (RVFV) is a zoonotic mosquito-transmitted arbovirus, presenting a serious threat to humans and animals. Susceptible hosts are of great significance for the prevention of RVFV. Appropriate animal models are helpful to better understand the onset and development of diseases, as well as the control measures and vaccine research. This review focuses on the role of animal hosts in the maintenance of the virus, and summarizes the host range of RVFV. We list some common animal models in the process of RVFV research, which would provide some important insights into the prevention and treatment of RVFV, as well as the study of Rift Valley fever (RVF) pathogenesis and vaccines.

## 1. Introduction

Rift Valley fever phlebovirus (RVFV) was initially isolated from ruminants in the Rift Valley region of Kenya in 1930 (1), and since then, it has been known to cause periodic outbreaks in Africa. In September 2000, RVF spread to Saudi Arabia and Yemen through the trade of RVFV-infected animals. Subsequently, from 2007 to 2022, RVF outbreaks were reported in over 20 countries, including Tanzania, Kenya, South Africa, Madagascar, and Mauritania, spanning a period of 15 years.^1^

RVFV belongs to the *Phlebovirus* genus in the *Phenuiviridae* family of Bunyavirales. It is an enveloped virus with a spherical shape (2). Similar to other bunyaviruses, RVFV possesses a single-stranded RNA genome that consists of three segments: large (L), medium (M), and small (S). The L segment encodes RNA-dependent RNA polymerase, the M segment encodes structural glycoproteins Gn and Gc, and the S segment encodes nucleoprotein (N) and a non-structural protein called NSs (2). NSs is considered the main virulence factor, and its deletion results in decreased infectivity of RVFV (3). The interaction between NSs and the host general transcription factor IIH (TFIIH, a multiprotein complex involved in both eukaryotic transcription and DNA repair) plays a crucial role in RVFV virulence. TFIIH is composed of 10 subunits, which can be divided into two functional complexes: the core complex (XPB, XPD, p62, p52, p44, p34, and p8/TTD-A) and the CDK-activated kinase (CAK) complex (CDK 7, cyclin H, and MAT 1) (4). When TFIIH associates with the ΩXaV motif in NSs, p62 is degraded, leading to the inhibition of the interferon (IFN) response and enhancing RVFV virulence (5). The mechanism by which RVFV infection inhibits host RNA synthesis and evades viral immune responses involves the competitive binding between NSs and p44. This competition prevents the interaction of XPD (the natural partner of p44 in TFIIH) with p44, and NSs sequesters certain TFIIH subunits within nuclear filamentous structures, leading to the segregation of the XPB/p44 complex and inhibiting the assembly of TFIIH subunits (6). Additionally, RVFV-encoded NSs proteins impact cellular fluidity, cell shape, and cell–cell adhesion by targeting the expression of Abl2 and the host actin cytoskeleton, thereby contributing to RVFV pathogenesis (7).

RVFV is primarily transmitted among animals through mosquito bites. However, for humans, RVFV infection can occur through contact with the blood of infected animals, inhalation or exposure to viral particles, and consumption of raw meat from sick animals (8, 9). This virus causes significant damage to ruminant livestock, resulting in high mortality rates among young newborn animals, widespread abortion in pregnant animals, and severe liver damage, posing a significant threat to animal health (8, 10, 11). Throughout history, RVFV has inflicted substantial harm on animal husbandry. In humans, the initial symptoms of RVFV infection include fever, headache, muscle and joint pain, and in some cases, nausea and vomiting. Conjunctivitis and photophobia may also occur. Severe cases can lead to bleeding, encephalitis, hepatitis, permanent blindness, or even death (12). Although there have been no reported cases of human-to-human transmission of RVFV, it is still considered a highly dangerous zoonotic pathogen. Aedes and Culex mosquitoes are the primary vectors responsible for the transmission of this disease between animals, as documented in the literature (13).

Given the broad host range of RVFV and the diversity of infected cells, host proteins play a crucial role in RVFV infection across different cell types and species. Identifying the function of these host factors is essential for the development of effective antiviral therapeutics. A genome-wide CRISPR/Cas9 screen revealed that low-density lipoprotein receptor-associated protein 1 (LRP1) is a critical host factor for RVFV infection. Heat shock protein (Grp94) and receptor-associated protein (RAP) were also found to influence RVFV entry by regulating the expression and function of LRP1 (14). The biological significance of LRP1 in RVFV infection was further demonstrated by inhibiting its interaction, which prevented RVFV from entering target cells across various host species. Studies have shown significant homology between the LRP1 protein in certain livestock species, such as cattle, and humans (15), suggesting that LRP1 is highly conserved among different species and may have a consistent function. This could potentially explain why humans are susceptible to RVFV after coming into contact with infected animals. Furthermore, LRP1 is widely expressed, with higher levels observed in the liver, placenta, and brain, which correspond to major sites of disease manifestation during RVFV infection. This highlights the potential of LRP1 as a target for antiviral therapeutics. Interestingly, LRP1 also plays a significant role in Oropouche orthobunyavirus infection (16), suggesting its potential involvement in the host infection process of Bunyaviruses. Further research is needed to explore the precise mechanisms underlying this relationship.

The RVF epidemic has had a significant impact on animal husbandry in areas where the disease is endemic. Therefore, understanding the host range of RVFV is crucial for preventing RVF outbreaks. Additionally, there is an urgent need for the research and development of effective vaccines and therapeutic drugs. However, the occurrence and progression of RVFV-induced diseases in humans are complex. It is impractical to deeply explore the pathogenesis and efficacy of these diseases in patients, thus biomedical research often relies on animal models as an experimental basis for testing hypotheses. Currently, laboratory infection models are established through virus inoculation, inhalation, or aerosol infection (Table 1). Some experiments use footpad infections to simulate the transmission mode of mosquito bites under realistic conditions. Different studies employ varying infection methods based on their specific research objectives. Furthermore, the choice of animal models depends on the specific research purposes. This review provides a summary of the geographical distribution of natural hosts for RVFV and the application and pathological responses of different animal models. These models are suitable for studying various pathological consequences associated with RVFV infection.

**Table 1:** Animal models of RVFV and their pathological manifestations.

Order	Genus or Species	Disease features	Route of exposure	References
Rodent	Mouse	Hepatitis	FP	(17)
		Hepatitis/cerebritis	IP/SC/Aerosol	(18)
		Hepatitis/cerebritis	IP/SC	(19)
		Cerebritis	FP	(20)
		Cerebritis	Aerosol	(21)
	Rat	Hepatitis	IP/SC/Aerosol	(22)
		Hepatitis	IP/SC/Aerosol	(23)
		Hepatitis/cerebritis/eye lesions	Aerosol	(24)
		Hepatitis	IP	(25)
		Eye lesions	SC/Aerosol	(26)
	Gerbil	Cerebritis	SC	(27)
	Hamster	Hepatitis	Aerosol	(28)
		Hepatitis	SC	(29)
Non-human primates	Rhesus monkey	Hepatitis/cerebritis/hemorrhage/fever	IV/IM	(30)
		Hepatitis/fever	Aerosol/IV	(31)
	Marmoset	Hepatitis/haemorrhag/cerebritis/fever	IV/SC/IN	(32)
		Cerebritis/hemorrhage/fever	Aerosol	(33)
	African green monkeys (AGM)	Cerebritis/hemorrhage/fever	Aerosol	(33)
		Cerebritis/hepatitis/hemorrhage/fever	Aerosol	(34)
Ruminantia	Sheep	Hepatitis/abortion	SC	(35)
		Fever/abortion	Inoculated	(36)
	Goats	Fever/hepatitis	SC	(37)
		Viremia	IN/SC	(38)
	Calves	Cerebritis/hepatitis	SC	(10)
	Lambs	Fever/hepatitis	Aerosol	(28)
		Hepatitis	Inoculated	(36)
	Cattle	Hepatitis/fever	IN/ID/IN+ID+SC	(39)
Other mammalia	Ferret	Fever/cerebritis	ID/IN	(40)

SC, subcutaneous; IP, intraperitoneal; FP, footpad; ID, intradermal; IN, intranasal.

## 2. Natural hosts

**Figure 1:**
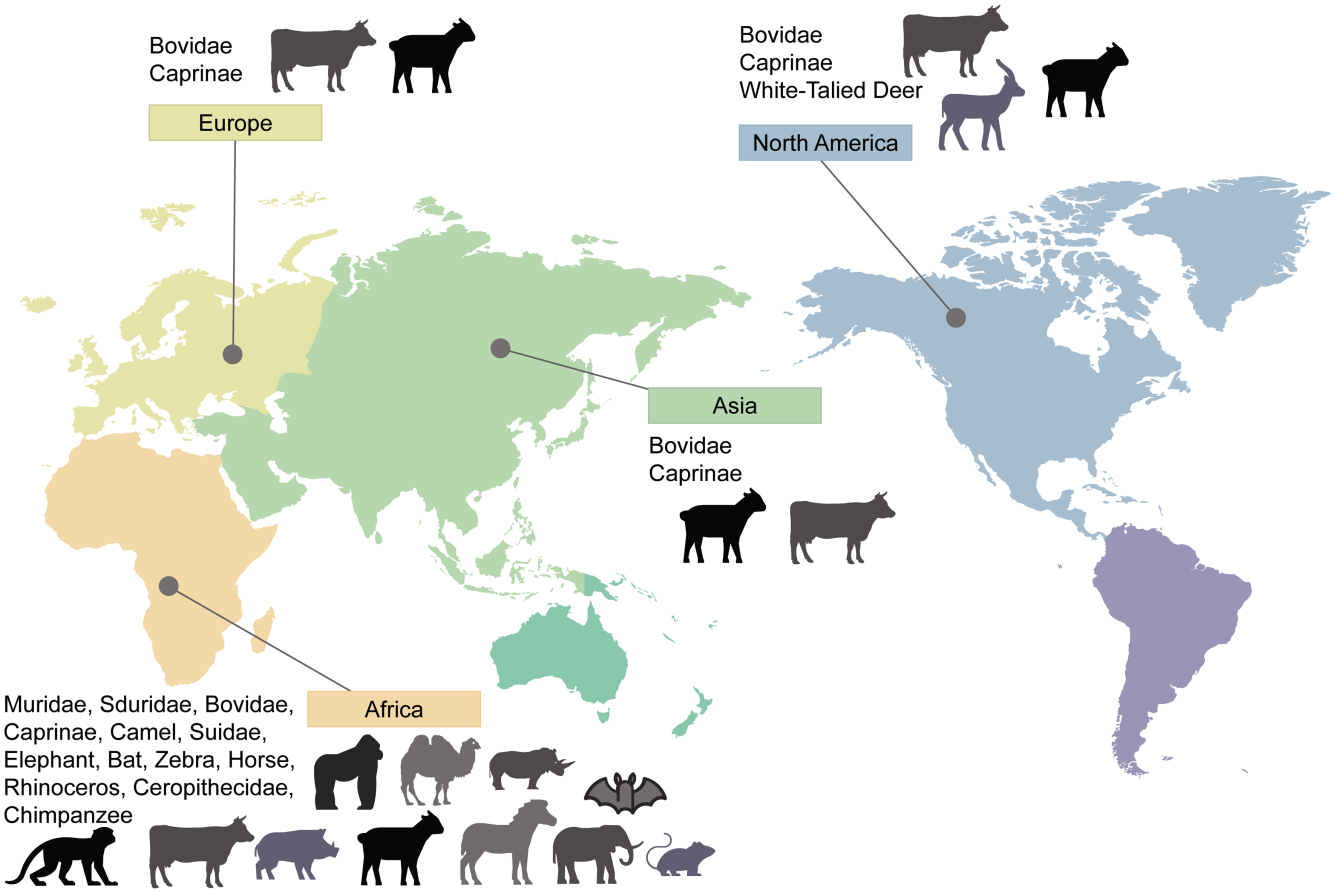
Worldwide distribution of RVFV hosts. Countries and areas at risk of RVFV transmission are based on reported cases of infected animals or corresponding research data that have demonstrated the risk of infection and their distribution. Please note that the absence of certain areas in the marking indicates that no study results were retrieved, and it does not necessarily imply the absence of the host in those areas.

As a zoonotic disease transmitted by animals, extensive research has been conducted on the natural hosts of RVFV. Due to common host factors, viruses have the ability to cross species barriers. The variation in organ damage severity may be attributed to the differential distribution of host factors in different organs. This allows RVFV to exhibit a broad host range and distinct manifestations of the disease. In this review, we have categorized them into rodents, ruminants, non-human primates, and other animals. To provide a visual representation, we have created a map showing the global distribution of RVFV animal hosts (Figure 1). The broad host range of RVFV, coupled with its mosquito-borne transmission characteristics, allows for viral mutation and sustained transmission over extended periods. Although the incidence of RVFV infection varies among different animal species, the detection rate of RVFV and the occurrence of viremia emphasize the crucial role of natural hosts in the prevalence and outbreak of RVFV.

### 2.1. Rodents

**Figure 2:**
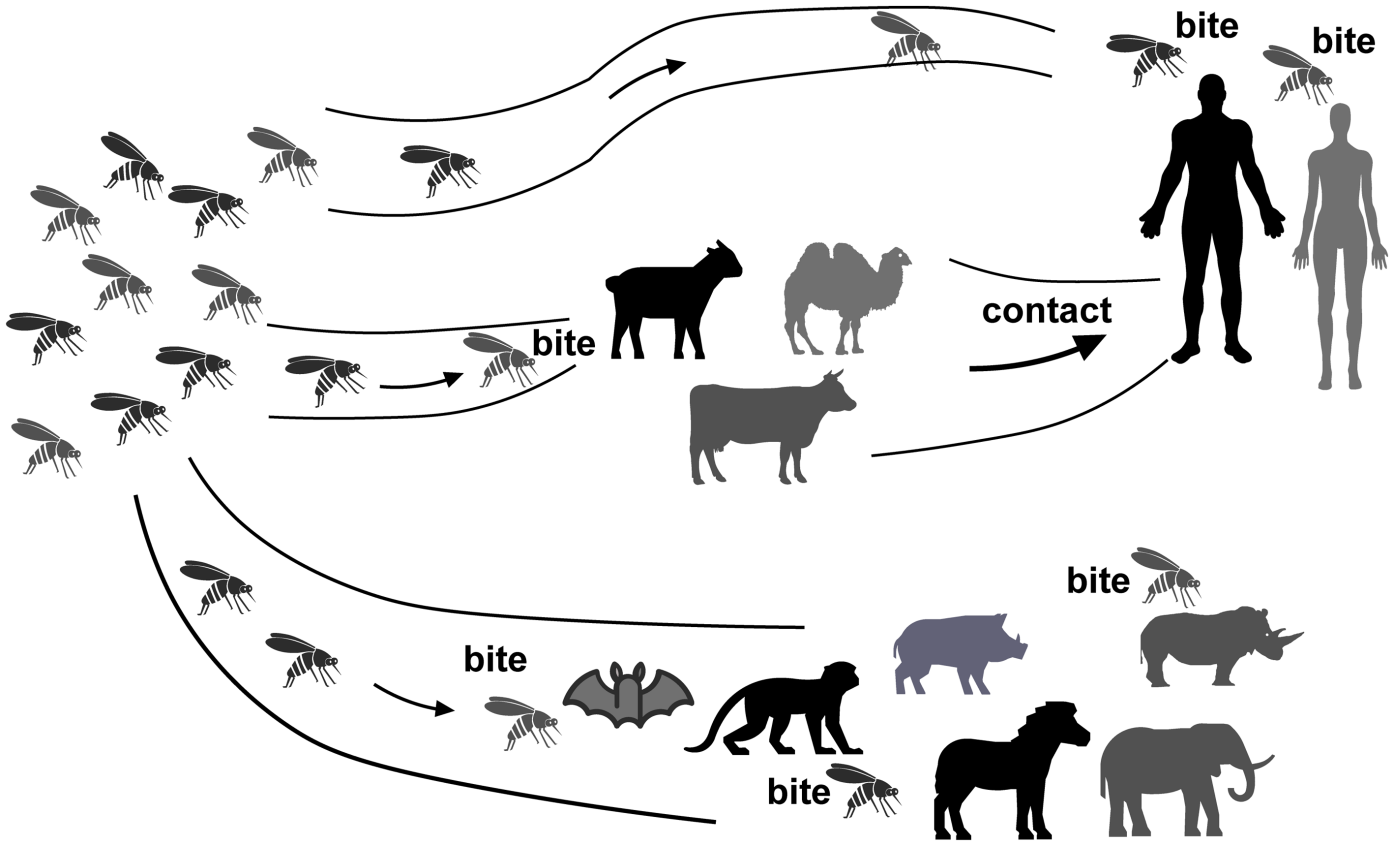
Transmission of RVFV between hosts. RVFV is transmitted to humans through mosquito bites, as mosquitoes become infected with the virus. Additionally, human exposure to infected animal blood or other bodily fluids can also contribute to the spread of RVFV.

While some studies suggest that wild rodents are not hosts for RVFV (41) most research findings indicate that wild rodents, being natural hosts, play a crucial role in the maintenance and transmission of RVFV. For instance, an ELISA test conducted in Egypt revealed a high RVFV positivity rate of 36.36% in rodents (42). Similarly, Senegal’s VNT test identified positive results in 4 out of the 14 rodent species examined, with the highest positivity rate observed in rodents from the low valley region of Senegal (43). It can be inferred that the variance in positivity rates is associated with the humidity levels in the respective regions where these species are found. This correlation is also evident from an ELISA survey conducted in South Africa, which showed an increased rate of rodent infection following heavy rainfall (44). In addition to the wide variety of rodents susceptible to RVFV, their early sexual maturity, rapid reproduction due to large litter sizes, and their ability to inhabit areas where humans or livestock reside make them potential risk factors for the further spread of RVFV transmission. HI testing conducted in the Sinai Peninsula has demonstrated RVFV infection among both rodents and local soldiers (45). As human populations expand, areas with lower living standards may struggle to maintain adequate health conditions, leading to increased contact between humans, livestock, and rodents, thereby facilitating virus transmission. A PCR test conducted in Egypt revealed significantly higher RVFV-positive rates among rats in rural areas compared to urban areas (46), indicating an extremely high risk of infection among local rural residents. Therefore, in RVFV-endemic regions, in addition to mosquito control and treatment of sick animals, controlling rodents is also crucial. Recent experimental infections have shown that black rats, despite not exhibiting any pathological manifestations following subcutaneous infection, develop antibodies and experience long-term viremia (47). Another investigation indicated that a higher concentration of the virus in the blood facilitates its spread (48), suggesting that this commonly found rodent species also poses a risk as a host for RVFV. The extensive infection of rodents and their associated pathological reactions have paved the way for rodents to be used as animal models in the development of RVFV vaccines and the exploration of pathogenesis (Figure 2).

### 2.2. Ruminants

Ruminants serve as the primary reservoir of RVFV and have a significant impact on economic development following RVFV infection. During the RVFV epidemic in Kenya, ruminants had much higher positivity rates in both antibody tests conducted compared to other wild animals (49, 50). The virus was first isolated from an infected flock of sheep during the 1930 RVF outbreak in East Africa (1). Various factors such as species differentiation, age of infection, and climatic conditions can affect the manifestation of the disease in ruminants. Pregnant female ruminants are particularly prone to having a “miscarriage storm” after being infected with RVFV. This suggests that the virus can cross the placenta and cause an increase in inflammatory chemokines and interferon response, leading to miscarriage.

Female ruminants were found to be more susceptible than males during RVFV outbreaks, as shown by antibody testing of rural buffalo in South Africa and PCR testing of cattle in Rwanda (51, 52). However, the exact cause of this gender discrepancy is uncertain. RVFV epidemics typically occur in autumn and winter, especially following heavy rains and floods, which create humid conditions that promote mosquito breeding.

Ruminant reproduction plays a significant role in determining the likelihood of a large-scale outbreak following infection, which in turn increases the risk of human infections. A Tanzanian study found that animal infection rates were closely related to human infection rates during epidemics, possibly due to increased local cattle slaughter, which is proportional to the human population (53). RVFV induces different pathologies in ruminants of different species, with cattle having a higher IgG positivity rate than sheep and goats in Cameroon. This may be due to feeding methods as nomadic cattle-raising methods increase the risk of cattle exposure to the virus.

IgM-positive samples and samples with successful RVFV RNA detected by PCR were only found in sheep and cattle, suggesting that goats may have lower susceptibility to the virus (54). A Tunisian survey showed higher antibody positivity rates in cattle and sheep than goats, providing further evidence for this speculation (55). In order to improve the specificity and sensitivity of detection, Gn-based ELISA methods are being increasingly applied to investigations (56). However, studies have shown that viremia peaks 3–4 days after infection in goats to activate innate immunity, and later produces neutralizing antibodies for long-term protection. This indicates that goats also play a role in RVFV maintenance (37).

Animal vaccination programmes are already in place in some areas, and surveys in Egypt have shown lower virus prevalence rates on vaccinated farms (57). However, a sample survey conducted in Rwanda following the implementation of the vaccine programme showed that no animals had been vaccinated, indicating the need for increased vaccine coverage (51). Authorities also need to pay attention to the risk of re-infection by other viruses to avoid adding to the burdens of infected animals (51).

Since researchers re-isolated the virus from camels in 1979, serological studies have shown that camels are susceptible hosts of RVFV (58). Additionally, serological studies of wild ruminants such as giraffes, antelopes, and buffalo have shown their overall susceptibility to RVFV, even though reported symptoms of the virus pandemic in wild ruminants are rare. It is likely that RVFV causes mild subclinical diseases in wild ruminants and maintains long-term low viremia, allowing the virus to continue to spread (50). Three separate ELISA surveys have revealed that the prevalence range of RVFV has expanded to the Middle East through ruminants (Table 2) (60–62). The high positive rate of RVFV detection in ruminants, as indicated by the EFSA Panel on Animal Health and Welfare (AHAW) PCR assay in 2020 on Mayot Island, further demonstrates the effectiveness and severity of RVFV infection in this group of animals. The region is under the jurisdiction of France and has closer trade ties with Europe, which has prompted European countries to be vigilant about the risks associated with the movement of goods (71). Although the RVFV gene is highly conserved, studies have identified some mutated strains in ruminants (72), indicating that ruminants also play a role in the evolution of the virus. This poses challenges in controlling its spread. Therefore, it is crucial for all countries to strengthen animal quarantine efforts at ports in order to effectively prevent the substantial losses that can be caused by the introduction of RVFV.

**Table 2:** Summary of animal hosts for RVFV.

Order	Family	Detection method	Location	References
Rodent	Muridae	HI	Zimbabwe, Egypt, Egypt	(41, 45, 48)
		VNT	Senegal	(43)
		ELISA	Egypt, South Africa	(42, 44)
		PCR	Egypt	(46)
	Sciuridae	VNT	Senegal	(43)
Ruminantia	Bovidae	HI	South Africa	(52)
		VNT	Kenya	(50)
		ELISA	Tanzania, Cameroon, Tunisia, Cameroon	(54, 55, 59)
		PCR	Egypt, Rwanda, Cameroon	(51, 54, 57)
	Caprinae	Virus Isolation	East Africa	(1)
		ELISA	Tunisia, Tanzania, Cameroon, Iran, Iraqi, Saudi Arabia	(53–55, 60–62)
		VNT	Mozambique, Senegal, Uganda, Yemen	(56)
		PCR	Zambia, Uganda, Cameroon, The Democratic Republic of the Congo	(54, 63–65)
	Camelidae	ELISA	Kenya	(49)
		Virus Isolation	Egypt	(58)
Mammalia	Suidae	VNT	South Africa	(66)
	Elephantidae	VNT	Kenya	(50)
	Vespertilionidae	Virus isolation	Guinea	(67)
		PCR	Egypt	(68)
	Equidae	VNT	Kenya	(50)
		Virus isolation	Egypt	(58)
	Rhinoceros	HI	Zimbabwe	(69)
		VNT	Kenya	(50)
Non-human primates	Cercopithecidae	/	Ghana to Angola	(70)
	Chimpanzee	/	Disjunct distribution in western and central Africa	(70)

HI, hemagglutination inhibition; VNT, virus neutralization test; ELISA, enzyme-linked immunosorbent assay; PCR, polymerase chain reaction.

### 2.3. Non-human primates

Due to limitations in field investigations, studies on primates have primarily been conducted in laboratory settings. It was only in 1954 that the presence of RVFV positivity in macaques was discovered during field investigations (70). Laboratory studies have focused on the manifestations of RVFV infection in red monkeys, long-tailed monkeys, white-eyebrowed monkeys, and rhesus monkeys. It was found that rhesus monkeys could develop viremia for up to 12 days after RVFV infection. Baboons infected with RVFV developed fever and viremia for several days (73). Intravenous inoculation of RVFV typically leads to benign viral infections in most rhesus monkeys. However, approximately 20% of cases still develop a hemorrhagic fever syndrome, characterized by extensive hepatic necrosis, disseminated intravascular coagulation, and hemolytic anemia (30). Another experimental infection of rhesus monkeys demonstrated that although all monkeys exhibited high levels of viral infection, the disease manifestations varied. A small proportion of monkeys infected with RVFV showed signs of hemorrhagic fever and eventually died. The remaining animals survived RVFV infection, but some displayed clinical symptoms such as loss of appetite and skin petechiae, while others showed no signs of clinical disease. In deceased macaques, abnormal liver function and coagulation markers were observed early in the infection, while monkeys without clinical manifestations exhibited high levels of IFN, suggesting that early morbidity events are critical factors for survival (74). The genes of non-human primates and humans exhibit a high degree of homology and similarity in terms of morphology and function. As hosts of RVFV, non-human primates serve as an alert for potential infections in humans. Therefore, during virus epidemics, the possibility of human infection should be taken into consideration.

### 2.4. Other animals

The mammalian host range of RVFV extends beyond ruminants and rodents. Serological tests have shown positive results for pigs and warthogs (66). RVFV has also been isolated from horses (58). In 1987, the virus was isolated from bats in Guinea (67), and in 2021, RVFV infection in bats was identified through PCR testing in Egypt (68). RVFV has been detected in rhinoceros (69), and serological investigations and PCR detection have revealed the infection of zebra, elephants, and rhinos with RVFV (50). A special investigation in 1996 focused on the RVFV positivity rate in carnivorous mammals such as jackals, wild dogs, cheetahs, and lions, and it was found that these animals could serve as natural hosts of RVFV (75). There have been no reported cases of RVFV infections in domestic pets, such as cats and dogs. However, given that many mammals are susceptible to RVFV, it remains uncertain whether domestic pets may play a role in the spread of the virus. Therefore, it is still important to monitor the infection status of pets. A laboratory study conducted in 2018 on North American white-tailed deer found that RVFV infection in these deer was associated with fever, hemorrhagic hepatic necrosis, and moderate to severe hemorrhagic lymphadenopathy, similar to the situation observed in ruminants. However, further attention and study are needed to understand the specific lesions, particularly moderate to severe diffuse hemorrhagic enteritis (76).

In addition to the natural hosts mentioned earlier, there have been studies exploring potential candidates as hosts for RVFV. Some vector mosquitoes of RVFV have been found to feed on amphibian blood (77), and *in vitro* studies have demonstrated the sensitivity of Xenopus cells and certain reptile cells to RVFV (78, 79). This suggests that amphibians or reptiles may potentially serve as natural hosts for RVFV. However, it is important to note that although RVFV infection in amphibians and reptiles has not been observed in their natural state, these animals are still at risk of RVFV infection, highlighting the broad host range of the virus.

## 3. Animal models

Considering the wide range of hosts for RVFV and the potential risk of transmission, further studies are necessary. However, it is important to note that different animal models and inoculation routes are suitable for studying diverse pathological processes related to RVFV. Each animal model possesses its own unique characteristics and advantages. Additionally, changes in hemogram parameters in each animal model are also worth considering (Table 1). As a reference, a summary of animal models for RVFV is provided (Table 1), which investigators can use to identify and select appropriate models based on their experimental requirements.

### 3.1. Rodents

Rodents are commonly used as animal models to study RVFV. Among rodents, rats, mice, and gerbils are the three main categories of animal models used. Gerbils, in particular, have been valuable in the study of neurological pathogenesis and can serve as effective animal models (27). However, it is important to note that the diversity of pathological changes observed in rodents following RVFV infection suggests that relying solely on a single rodent species for research may not be sufficient to ensure the reliability of experimental results or meet all experimental requirements.

Rodents are primarily infected with RVFV through direct intranasal injection or aerosol infection (18, 19, 21, 25, 28) (Table 1), as they are intranasally susceptible to the virus. Intraperitoneal injection can also lead to successful infection, but this method differs from the natural transmission route, which involves mosquito bites or contact with contaminated tissues. To simulate the natural infection process and study the status of human infection under more realistic conditions, recent experiments have utilized footpad infection in rodents (17). Different strains of animals exhibit varying pathological changes following RVFV infection, with most rats and mice being suitable for studying liver-related injuries. However, the most severe reactions observed in humans after RVFV infection involve central nervous system (CNS) lesions and permanent blindness caused by ocular lesions. Consequently, efforts have been made to develop rat and mouse models that can be used to study these specific aspects. For instance, Haley Cartwright et al. found that CC057 strain mice infected *via* footpad can be used to study encephalitis (20). Madeline M. Schwarz et al. demonstrated the tropism of RVFV for Lrp1 in the posterior eye of Sprague Dawley rats, making them suitable for studying uvea, retina, and optic nerve damage (26). Additionally, pregnant rats have been found to be more susceptible to RVFV infection compared to non-pregnant rats. RVFV infection in pregnant rats can lead to intrauterine fetal death and severe congenital abnormalities. During the second trimester, RVFV can directly infect placental chorionic villi in human placental tissue. Pregnant rats can transmit RVFV directly and vertically through the placenta, making them suitable models for studying RVFV-induced abortion, which closely mimics the situation in pregnant humans (80). During the development of animal models, researchers discovered that hamsters can be utilized as an animal model for studying liver lesions caused by RVFV infection (29). This finding further expands the host range of RVFV in rodents. The use of hamsters as an animal model for RVFV research dates back to as early as 1962. Subcutaneous infection in hamsters leads to noticeable clinical symptoms, indicating that hamsters can be effectively employed in the study of RVFV, similar to other rodent models.

### 3.2. Ruminants

Due to the significant role of ruminants in the maintenance of RVFV, these animals serve as ideal animal models in vaccine studies. Ruminants, such as sheep, goats, and cattle, are commonly used in the development of animal vaccines against RVFV. Given the effects of RVFV on pregnant ruminants, such as liver necrosis and abortion (8), pregnant ewes or cows are often included as separate populations to assess the application range and effects of vaccines during the research and development process. Furthermore, the efficacy of RVFV infection differs among animals of different ages. Newborn sheep or calves are more susceptible to RVFV, emphasizing the need to consider the efficacy and safety of vaccines for both the young animals and their parents, who may be infected with RVFV. Researchers have investigated the efficacy and safety of the four-segmented RVFV (RVFV-4s) vaccine in young sheep, goats, and cattle (11). Additionally, the efficacy of the nonspreading RVFV (NSR) vaccine has been studied specifically in lambs (81). With the vRVFV-4s vaccine, transmission of the virus is not observed in vaccinated animals or in the environment, and the virus does not regain virulence upon animal passage. This vaccine has proven effective in protecting various ruminant species from their corresponding RVFV strains (11). It provides some relief from liver damage in infected pregnant animals and reduces the risk of miscarriage caused by viral infection (36). However, there are notable variations among different species, and the immune response in young sheep and cattle is not entirely satisfactory. Similar to vRVFV-4s, MP-12 does not exhibit viral shedding or transmission (82). However, its use during early pregnancy may lead to partial abortion (83). NSR can reduce viremia in lambs to a level undetectable by viral isolation, thereby protecting them from clinical symptoms, although this effect is not long-lasting. High-precision detection has shown that RVFV can also be transmitted from pregnant ewes to their fetuses, indicating that the vaccine’s immunization efficacy has not met expectations. Different inoculation methods in ruminants result in varying clinical manifestations. In calves, natural RVFV infection primarily leads to liver lesions, while subcutaneous infection tests have revealed encephalomyelitis, lymphatic necrosis, and adrenal gland damage (10). Studies on different routes of infection in cattle and goats have indicated that intranasal infection is more likely to cause neurological damage (38, 39). Neuronal infection in goats has been observed as early as 1 day after infection (38). Although short-term infection is unlikely to be attributed to high levels of viremia breaking through the blood–brain barrier, it is plausible that intranasal infection directly affects neurons. This finding offers a viable direction for future research on neurological lesions caused by RVFV. Furthermore, the immune status of animals after virus infection, including changes in interferon levels, pro-inflammatory factors, antibodies, etc., also influences clinical symptoms and should be given due attention (37). Table 1 provides an overview of the main clinical manifestations and experimental infection routes observed in these ruminant animal models. This information can serve as a valuable reference for future development of animal vaccines.

### 3.3. Non-human primates

Non-human primates are used as animal models to study the harm of RVFV to the human body due to their high similarity to humans in terms of pathogenesis and clinical manifestations. These animal models serve as valuable tools in the development of vaccines for human use. Despite being expensive and challenging to obtain approval for their use, non-human primates are still essential in studying RVFV-induced neurological diseases. This is because stable encephalitis models are not commonly observed in rodents such as rats, mice, and gerbils, primarily due to age limitations. Rhesus monkeys, as long-term non-human primate models widely used in various viral studies, exhibit phenotypic similarities to humans after RVFV infection. However, due to the low incidence of neurological diseases in rhesus monkeys, they may not be the most suitable models for RVFV studies (30, 31). Studies have shown that African green monkeys and marmosets demonstrate more significant clinical manifestations, including neurological symptoms, when establishing RVFV infection models (32–34). In non-human primates, the varying disease manifestations observed after infection may be attributed to differences in host defense status and the distribution of host factors. This finding holds significant implications for humans as well. Therefore, these non-human primate species are considered more suitable for relevant RVFV studies as animal models (Table 1).

### 3.4. Other animals

Recently, ferrets have emerged as a potential animal model for studying RVFV. When inoculated intranasally with RVFV, ferrets have shown a high likelihood of developing central nervous system (CNS) diseases, characterized by symptoms such as seizures and ataxia (40). This model is particularly valuable because the RVFV-induced CNS diseases observed in ferrets occur following exposure, thereby mimicking the natural exposure pathway seen in humans. Consequently, the RVFV ferret model can be utilized to investigate how the virus enters the CNS. In addition, ferrets can also serve as animal models for studying mild self-limited febrile illness caused by RVFV. Although this may not be the most severe symptom, it is still important to consider, as it represents a significant manifestation of human RVFV infection and warrants attention. Therefore, ferrets provide a valuable tool for studying both the CNS effects and mild febrile illness associated with RVFV.

## 4. Conclusion and prospect

The host range of RVFV infection is determined by host receptors and entry factors. It has been discovered that human LRP1 serves as a receptor for RVFV. However, the conservation of LRP1 protein sequence is relatively low in humans and some RVFV-sensitive animals, suggesting the existence of other receptors in these animals. In mice, sheep, and *Aedes aegypti*, homologs of C-type lectin receptors (CLRs) have been proposed as potential attachment factors or entry receptors in various species (84).

The heterogeneity among hosts in RVFV infection can be attributed to factors such as the efficiency of viral replication in the host and the survival time of infectious viral particles. Studies on host resistance to RVFV have revealed that the viral glycoprotein Gn plays a significant role in triggering immune responses on the surface of the RVFV viral envelope. Gn-specific antibodies are a major component of the RVFV neutralizing antibody response, indicating that the entry of RVFV into the host depends on Gn (85). Therefore, these two critical molecules, Gn and LRP1, can be potential targets for future vaccines and drug development. In addition to inhibiting viral entry, translational arrest and autophagy are also considered integral components of host defense against RVFV (86, 87). Cholesterol can be incorporated into RVFV particles and enhance RVFV infectivity in a polyamine-dependent manner (88). Studies have found that a high-cholesterol diet can lead to liver cholesterol accumulation, and it mainly affects cerebral vessels among the vascular effects, with up-regulation of LDLr and LRP1 detected in cerebral vessels (89). This finding aligns with the liver and brain lesions caused by RVFV infection, but the specific relationship still requires further exploration.

Furthermore, local ecological factors, such as the relative abundance and feeding preferences of vector hosts, can influence the transmission of RVFV among hosts. The prevalence of RVFV is closely linked to ecological and climatic conditions (90). Mosquito species in North America and European Aedes mosquitoes have been found to be capable of infecting and transmitting RVFV (91–95). With the impacts of climate change and global trade, these mosquitoes have the potential to spread the virus to Europe and the Americas, posing a significant risk to animal husbandry. Therefore, it is crucial to address and mitigate this risk to prevent irreversible damage. The variation in pathological responses to RVFV infection among different host species is an important consideration in selecting animal models for research. The US Food and Drug Administration recommends testing potential vaccines and treatments in at least two well-established animal models (96). Multiple animal models have been utilized to confirm the efficacy and safety of the RVFV-4s and MP-12 vaccines.

In conclusion, this review highlights the risk of RVFV transmission by providing an overview of its host range. Although an inactivated vaccine has been developed, it has not yet been licensed for commercial use. Currently, the vaccine is only administered to protect veterinarians and laboratory personnel who may be at high risk of exposure to RVFV. However, the infectivity of RVFV to humans and its potential to cause severe illness or even death cannot be ignored. Therefore, there is still much research needed in the prevention and treatment of RVFV. To provide a reference for future research, this review summarizes the commonly used animal models in RVFV studies and emphasizes the pathological findings associated with RVFV infection in different host models. Presently, the available animal models for studying visual impairment and nervous system damage caused by RVFV are insufficient to meet the demands of scientific research. This poses challenges for the prevention and treatment of these two symptoms. Future model development should focus on these symptoms, adjust research directions, and address the gaps in understanding the immunopathology of such symptoms. Furthermore, it is worth noting that different hosts exhibit variations in their response to RVFV infection. For instance, some animals may develop ocular lesions while others can escape death resulting from liver damage but still experience severe encephalitis. The underlying reasons for these differences, including the distribution of RVFV host factors in various host animals or the existence of alternative antiviral pathways, require further investigation and exploration. Additionally, attention should be given to the similarities between RVFV and other hemorrhagic fever viruses. This includes examining whether there is cross-reactivity between factors involved in mediating the infection of each virus. Such investigations can shed light on the feasibility of combined prevention and treatment strategies.

## Author contributions

YX: Writing – original draft. XW: Writing – review & editing. LJ: Writing – review & editing. YZ: Writing – review & editing. YL: Software, Writing – review & editing. FW: Writing – review & editing. LZ: Conceptualization, Writing – review & editing.

## Footnotes

^1^ https://wahis.woah.org/#/event-management

## References

[1] Daubney R Hudson JR Garnham PC. Enzootic hepatitis or Rift Valley fever. an undescribed virus disease of sheep cattle and man from East Africa. J Pathol Bacteriol. (1931) 34:545–79.

[2] Wright D Kortekaas J Bowden TA Warimwe GM. Rift Valley fever: biology and epidemiology. J Gen Virol. (2019) 100:1187–99. doi: 10.1099/jgv.0.001296 31310198 PMC7613496

[3] Muller R Saluzzo JF Lopez N Dreier T Turell M Smith J. Characterization of clone 13, a naturally attenuated avirulent isolate of Rift Valley fever virus, which is altered in the small segment. Am J Trop Med Hygiene. (1995) 53:405–11. doi: 10.4269/ajtmh.1995.53.405, PMID: 7485695

[4] Kolesnikova O Radu L Poterszman A. TFIIH: a multi-subunit complex at the cross-roads of transcription and DNA repair. Adv Protein Chem Struct Biol. (2019) 115:21–67. doi: 10.1016/bs.apcsb.2019.01.003, PMID: 30798933

[5] Cyr N de la Fuente C Lecoq L Guendel I Chabot PR Kehn-Hall K. A ΩXaV motif in the Rift Valley fever virus NSs protein is essential for degrading p 62, forming nuclear filaments and virulence. Proc Natl Acad Sci U S A. (2015) 112:6021–6. doi: 10.1073/pnas.1503688112, PMID: 25918396 PMC4434773

[6] Le May N Dubaele S Proietti De Santis L Billecocq A Bouloy M Egly JM. TFIIH transcription factor, a target for the Rift Valley hemorrhagic fever virus. Cells. (2004) 116:541–50. doi: 10.1016/S0092-8674(04)00132-1, PMID: 14980221

[7] Bamia A Marcato V Boissière M Mansuroglu Z Tamietti C Romani M. The NSs protein encoded by the virulent strain of Rift Valley fever virus targets the expression of Abl 2 and the actin cytoskeleton of the host, affecting cell mobility, cell shape, and cell-cell adhesion. J Virol. (2020) 95:e01768-20. doi: 10.1128/JVI.01768-20, PMID: 33087469 PMC7737741

[8] Odendaal L Clift SJ Fosgate GT Davis AS. Ovine fetal and placental lesions and cellular tropism in natural Rift Valley fever virus infections. Vet Pathol. (2020) 57:791–806. doi: 10.1177/0300985820954549, PMID: 32885745

[9] LaBeaud AD Muchiri EM Ndzovu M Mwanje MT Muiruri S Peters CJ. Interepidemic Rift Valley fever virus seropositivity, northeastern Kenya. Emerg Infect Dis. (2008) 14:1240–6. doi: 10.3201/eid1408.080082, PMID: 18680647 PMC2600406

[10] Rippy MK Topper MJ Mebus CA Morrill JC. Rift Valley fever virus-induced encephalomyelitis and hepatitis in calves. Vet Pathol. (1992) 29:495–502. doi: 10.1177/030098589202900602, PMID: 1448895

[11] Wichgers Schreur PJ Oreshkova N van Keulen L Kant J van de Water S Soós P. Safety and efficacy of four-segmented Rift Valley fever virus in young sheep, goats and cattle. NPJ Vaccines. (2020) 5:65. doi: 10.1038/s41541-020-00212-4 32728479 PMC7382487

[12] Bird BH Ksiazek TG Nichol ST Maclachlan NJ. Rift Valley fever virus. J Am Vet Med Assoc. (2009) 234:883–93. doi: 10.2460/javma.234.7.883 19335238

[13] Linthicum KJ Britch SC Anyamba A. Rift Valley fever: an emerging mosquito-borne disease. Annu Rev Entomol. (2016) 61:395–415. doi: 10.1146/annurev-ento-010715-023819 26982443

[14] Ganaie SS Schwarz MM McMillen CM Price DA Feng AX Albe JR. Lrp 1 is a host entry factor for Rift Valley fever virus. Cells. (2021) 184:5163–5178.e24. doi: 10.1016/j.cell.2021.09.001 PMC8786218 34559985

[15] Bopp NE Fernández D Aguilar PV. Closing the rift: discovery of a novel virus receptor. Cells. (2021) 184:5084–6. doi: 10.1016/j.cell.2021.09.004, PMID: 34559984

[16] Schwarz MM Price DA Ganaie SS Feng A Mishra N Hoehl RM. Oropouche orthobunyavirus infection is mediated by the cellular host factor Lrp 1. Proc Natl Acad Sci U S A. (2022) 119:e2204706119. doi: 10.1073/pnas.2204706119, PMID: 35939689 PMC9388146

[17] Cartwright HN Barbeau DJ McElroy AK. Rift Valley fever virus is lethal in different inbred mouse strains independent of sex. Front Microbiol. (2020) 11:1962. doi: 10.3389/fmicb.2020.01962, PMID: 32973712 PMC7472459

[18] Smith DR Steele KE Shamblin J Honko A Johnson J Reed C. The pathogenesis of Rift Valley fever virus in the mouse model. Virology. (2010) 407:256–67. doi: 10.1016/j.virol.2010.08.016 20850165

[19] Batista L Jouvion G Simon-Chazottes D Houzelstein D Burlen-Defranoux O Boissière M. Genetic dissection of Rift Valley fever pathogenesis: Rvfs 2 locus on mouse chromosome 11 enables survival to early-onset hepatitis. Sci Rep. (2020) 10:8734. doi: 10.1038/s41598-020-65683-w, PMID: 32457349 PMC7250886

[20] Cartwright HN Barbeau DJ Doyle JD Klein E Heise MT Ferris MT. Genetic diversity of collaborative cross mice enables identification of novel rift valley fever virus encephalitis model. PLoS Pathog. (2022) 18:e1010649. doi: 10.1371/journal.ppat.1010649, PMID: 35834486 PMC9282606

[21] Reed C Lin K Wilhelmsen C Friedrich B Nalca A Keeney A. Aerosol exposure to Rift Valley fever virus causes earlier and more severe neuropathology in the murine model, which has important implications for therapeutic development. PLoS Negl Trop Dis. (2013) 7:e2156. doi: 10.1371/journal.pntd.0002156, PMID: 23593523 PMC3617210

[22] Peters CJ Slone TW. Inbred rat strains mimic the disparate human response to Rift Valley fever virus infection. J Med Virol. (1982) 10:45–54. doi: 10.1002/jmv.1890100107, PMID: 7130966

[23] Anderson GW Jr Smith JF. Immunoelectron microscopy of Rift Valley fever viral morphogenesis in primary rat hepatocytes. Virology. (1987) 161:91–100. doi: 10.1016/0042-6822(87)90174-7, PMID: 3499704

[24] Anderson GW Jr Lee JO Anderson AO Powell N Mangiafico JA Meadors G. Efficacy of a Rift Valley fever virus vaccine against an aerosol infection in rats. Vaccine. (1991) 9:710–4. doi: 10.1016/0264-410X(91)90285-E, PMID: 1759489

[25] Ritter M Bouloy M Vialat P Janzen C Haller O Frese M. Resistance to Rift Valley fever virus in *Rattus norvegicus*: genetic variability within certain 'inbred' strains. J Gen Virol. (2000) 81:2683–8. doi: 10.1099/0022-1317-81-11-2683, PMID: 11038380

[26] Schwarz MM Connors KA Davoli KA McMillen CM Albe JR Hoehl RM. Rift Valley fever virus infects the posterior segment of the eye and induces inflammation in a rat model of ocular disease. J Virol. (2022) 96:e0111222. doi: 10.1128/jvi.01112-22, PMID: 36194021 PMC9599513

[27] Anderson GW Jr Slone TW Jr Peters CJ. The gerbil, *Meriones unguiculatus*, a model for Rift Valley fever viral encephalitis. Arch Virol. (1988) 102:187–96. doi: 10.1007/BF01310824, PMID: 3060046

[28] Miller WS Demchak P Rosenberger CR Dominik JW Bradshaw JL. Stability and infectivity of airborne yellow fever and rift valley fever viruses. Am J Hyg. (1963) 77:114–21.

[29] Scharton D Van Wettere AJ Bailey KW Vest Z Westover JB Siddharthan V. Rift Valley fever virus infection in golden Syrian hamsters. PLoS One. (2015) 10:e0116722. doi: 10.1371/journal.pone.0116722, PMID: 25607955 PMC4301868

[30] Peters CJ Jones D Trotter R Donaldson J White J Stephen E. Experimental Rift Valley fever in rhesus macaques. Arch Virol. (1988) 99:31–44. doi: 10.1007/BF01311021, PMID: 3355374

[31] Morrill JC Peters CJ. Mucosal immunization of rhesus macaques with Rift Valley fever MP-12 vaccine. J Infect Dis. (2011) 204:617–25. doi: 10.1093/infdis/jir354, PMID: 21791664

[32] Smith DR Bird BH Lewis B Johnston SC McCarthy S Keeney A. Development of a novel nonhuman primate model for Rift Valley fever. J Virol. (2012) 86:2109–20. doi: 10.1128/JVI.06190-11, PMID: 22156530 PMC3302397

[33] Hartman AL Powell DS Bethel LM Caroline AL Schmid RJ Oury T. Aerosolized rift valley fever virus causes fatal encephalitis in African green monkeys and common marmosets. J Virol. (2014) 88:2235–45. doi: 10.1128/JVI.02341-13, PMID: 24335307 PMC3911574

[34] Wonderlich ER Caroline AL McMillen CM Walters AW Reed DS Barratt-Boyes SM. Peripheral blood biomarkers of disease outcome in a monkey model of Rift Valley fever encephalitis. J Virol. (2018) 92:e01662-17. doi: 10.1128/JVI.01662-17, PMID: 29118127 PMC5774883

[35] Yedloutschnig RJ Dardiri AH Mebus CA Walker JS. Abortion in vaccinated sheep and cattle after challenge with Rift Valley fever virus. Vet Rec. (1981) 109:383–4. doi: 10.1136/vr.109.17.383, PMID: 7340068

[36] Wichgers Schreur PJ Oymans J Kant J van de Water S Kollár A Dehon Y. A single vaccination with four-segmented rift valley fever virus prevents vertical transmission of the wild-type virus in pregnant ewes. NPJ Vaccines. (2021) 6:8. doi: 10.1038/s41541-020-00271-7, PMID: 33420095 PMC7794363

[37] Nfon CK Marszal P Zhang S Weingartl HM. Innate immune response to Rift Valley fever virus in goats. PLoS Negl Trop Dis. (2012) 6:e1623. doi: 10.1371/journal.pntd.0001623, PMID: 22545170 PMC3335883

[38] Kroeker AL Smid V Embury-Hyatt C Moffat E Collignon B Lung O. RVFV infection in goats by different routes of inoculation. Viruses. (2018) 10:709. doi: 10.3390/v10120709, PMID: 30545088 PMC6316315

[39] Kroeker AL Babiuk S Pickering BS Richt JA Wilson WC. Livestock challenge models of Rift Valley fever for agricultural vaccine testing. Front Vet Sci. (2020) 7:238. doi: 10.3389/fvets.2020.00238, PMID: 32528981 PMC7266933

[40] Barbeau DJ Albe JR Nambulli S Tilston-Lunel NL Hartman AL Lakdawala SS. Rift Valley fever virus infection causes acute encephalitis in the ferret. mSphere. (2020) 5:e00798-20. doi: 10.1128/mSphere.00798-20 PMC7593599 33115835

[41] Hoogstraal H Meegan JM Khalil GM Adham FK. The Rift Valley fever epizootic in Egypt 1977-78. 2. Ecological and entomological studies. Trans R Soc Trop Med Hyg. (1979) 73:624–9. doi: 10.1016/0035-9203(79)90005-1, PMID: 44038

[42] Youssef BZ Donia HA. The potential role of *Rattus rattus* in enzootic cycle of Rift Valley fever in Egypt. 1-detection of RVF antibodies in *R. rattus* blood samples by both enzyme linked immuno sorbent assay (ELISA) and immuno-diffusion technique (ID). J Egypt Public Health Assoc. (2001) 76:431–41. PMID: 17216936

[43] Gora D Yaya T Jocelyn T Didier F Maoulouth D Amadou S. The potential role of rodents in the enzootic cycle of Rift Valley fever virus in Senegal. Microbes Infect. (2000) 2:343–6. doi: 10.1016/S1286-4579(00)00334-8, PMID: 10817634

[44] Pretorius A Oelofsen MJ Smith MS van der Ryst E. Rift Valley fever virus: a seroepidemiologic study of small terrestrial vertebrates in South Africa. Am J Trop Med Hygiene. (1997) 57:693–8. doi: 10.4269/ajtmh.1997.57.693, PMID: 9430529

[45] Kark JD Aynor Y Ben Mordechai Y Ron-Kuper N Peleg BA Peters CJ. A serological survey of Rift Valley fever antibodies in the northern Sinai. Trans R Soc Trop Med Hyg. (1982) 76:427–30. doi: 10.1016/0035-9203(82)90129-8, PMID: 6926758

[46] Youssef BZ Donia HA. The potential role of *rattus rattus* in enzootic cycle of Rift Valley fever in Egypt 2-application of reverse transcriptase polymerase chain reaction (RT-PCR) in blood samples of *Rattus rattus*. J Egypt Public Health Assoc. (2002) 77:133–41. PMID: 17219894

[47] Stoek F Rissmann M Ulrich R Eiden M Groschup MH. Black rats (*Rattus rattus*) as potential reservoir hosts for Rift Valley fever phlebovirus: experimental infection results in viral replication and shedding without clinical manifestation. Transbound Emerg Dis. (2022) 69:1307–18. doi: 10.1111/tbed.14093, PMID: 33794070

[48] Swanepoel R Blackburn NK Efstratiou S Condy JB. Studies on Rift Valley fever in some African murids (Rodentia: Muridae). J Hyg. (1978) 80:183–96. doi: 10.1017/S0022172400053535, PMID: 632561 PMC2130003

[49] Britch SC Binepal YS Ruder MG Kariithi HM Linthicum KJ Anyamba A. Rift Valley fever risk map model and seroprevalence in selected wild ungulates and camels from Kenya. PLoS One. (2013) 8:e66626. doi: 10.1371/journal.pone.0066626, PMID: 23840512 PMC3695998

[50] Evans A Gakuya F Paweska JT Rostal M Akoolo L Van Vuren PJ. Prevalence of antibodies against Rift Valley fever virus in Kenyan wildlife. Epidemiol Infect. (2008) 136:1261–9. doi: 10.1017/S0950268807009806, PMID: 17988425 PMC2870911

[51] Dutuze MF Ingabire A Gafarasi I Uwituze S Nzayirambaho M Christofferson RC. Identification of Bunyamwera and possible other Orthobunyavirus infections and disease in cattle during a Rift Valley fever outbreak in Rwanda in 2018. Am J Trop Med Hyg. (2020) 103:183–9. doi: 10.4269/ajtmh.19-0596, PMID: 32314686 PMC7356447

[52] LaBeaud AD Cross PC Getz WM Glinka A King CH. Rift Valley fever virus infection in African buffalo (*Syncerus caffer*) herds in rural South Africa: evidence of interepidemic transmission. Am J Trop Med Hyg. (2011) 84:641–6. doi: 10.4269/ajtmh.2011.10-0187, PMID: 21460024 PMC3062463

[53] Sindato C Karimuribo ED Vairo F Misinzo G Rweyemamu MM Hamid MMA. Rift Valley fever seropositivity in humans and domestic ruminants and associated risk factors in Sengerema, Ilala, and Rufiji districts, Tanzania. Int J Infect Dis. (2022) 122:559–65. doi: 10.1016/j.ijid.2022.07.012, PMID: 35811085

[54] Sado FY Tchetgna HS Kamgang B Djonabaye D Nakouné E McCall PJ. Seroprevalence of Rift Valley fever virus in domestic ruminants of various origins in two markets of Yaoundé, Cameroon. PLoS Neglect Trop Dis. (2022) 16:e0010683. doi: 10.1371/journal.pntd.0010683, PMID: 35951644 PMC9397978

[55] Zouaghi K Bouattour A Aounallah H Surtees R Krause E Michel J. First serological evidence of Crimean-Congo hemorrhagic fever virus and Rift Valley fever virus in ruminants in Tunisia. Pathogens. (2021) 10:769. doi: 10.3390/pathogens10060769 34207423 PMC8234966

[56] Jäckel S Eiden M Balkema-Buschmann A Ziller M van Vuren PJ Paweska JT. A novel indirect ELISA based on glycoprotein Gn for the detection of IgG antibodies against Rift Valley fever virus in small ruminants. Res Vet Sci. (2013) 95:725–30. doi: 10.1016/j.rvsc.2013.04.015, PMID: 23664015

[57] Mroz C Gwida M El-Ashker M Ziegler U Homeier-Bachmann T Eiden M. Rift Valley fever virus infections in Egyptian cattle and their prevention. Transbound Emerg Dis. (2017) 64:2049–58. doi: 10.1111/tbed.12616, PMID: 28116860

[58] Imam IZ El-Karamany R Darwish MA. An epidemic of Rift Valley fever in Egypt. 2. Isolation of the virus from animals. Bull World Health Organ. (1979) 57:441–3. PMID: 314355 PMC2395804

[59] Bronsvoort BM Kelly RF Freeman E Callaby R Bagninbom JM Ndip L. Population-based, seroepidemiological study of Rift Valley fever in Cameroonian cattle populations. Front Vet Sci. (2022) 9:897481. doi: 10.3389/fvets.2022.897481, PMID: 35774979 PMC9237551

[60] Aghaa OB Rhaymah MS. Seroprevelance study of Rift Valley fever antibody in sheep and goats in Ninevah governorate. Iraqi J Vet Sci. (2013) 27:53–61. doi: 10.33899/IJVS.2013.82778

[61] Al-Afaleq AI Hussein MF Al-Naeem AA Housawi F Kabati AG. Seroepidemiological study of Rift Valley fever (RVF) in animals in Saudi Arabia. Trop Anim Health Prod. (2012) 44:1535–9. doi: 10.1007/s11250-012-0100-x, PMID: 22359088

[62] Fakour S Naserabadi S Ahmadi E. A serological and hematological study on rift valley fever and associated risk factors in aborted sheep at Kurdistan province in west of Iran. Comp Immunol Microbiol Infect Dis. (2021) 75:101620. doi: 10.1016/j.cimid.2021.101620, PMID: 33609990

[63] Tshilenge GM Mulumba MLK Misinzo G Noad R Dundon WG. Rift Valley fever virus in small ruminants in the Democratic Republic of the Congo. Onderstepoort J Vet Res. (2019) 86:e1–5. doi: 10.4102/ojvr.v86i1.1737, PMID: 31714136 PMC6852419

[64] Shoemaker TR Nyakarahuka L Balinandi S Ojwang J Tumusiime A Mulei S. First laboratory-confirmed outbreak of human and animal Rift Valley fever virus in Uganda in 48 years. Am J Trop Med Hyg. (2019) 100:659–71. doi: 10.4269/ajtmh.18-0732, PMID: 30675833 PMC6402942

[65] Chambaro HM Hirose K Sasaki M Libanda B Sinkala Y Fandamu P. An unusually long rift valley fever inter-epizootic period in Zambia: evidence for enzootic virus circulation and risk for disease outbreak. PLoS Negl Trop Dis. (2022) 16:e0010420. doi: 10.1371/journal.pntd.0010420, PMID: 35653390 PMC9197056

[66] Lubisi BA Ndouvhada PN Neiffer D Penrith ML Sibanda DR Bastos ADS. Evaluation of a virus neutralisation test for detection of Rift Valley fever antibodies in Suid sera. Trop Med Infect Dis. (2019) 4:52. doi: 10.3390/tropicalmed4010052, PMID: 30934604 PMC6473580

[67] Boiro I Konstaninov OK Numerov AD. Isolation of Rift Valley fever virus from bats in the Republic of Guinea. Bull Soc Pathol Exotique Filiales. (1987) 80:62–7. PMID: 3607999

[68] Saeed OS El-Deeb AH Gadalla MR El-Soally SAG Ahmed HAH. Genetic characterization of Rift Valley fever virus in insectivorous bats, Egypt. Vector Borne Zoonotic Dis. (2021) 21:1003–6. doi: 10.1089/vbz.2021.0054. 34958267

[69] Anderson EC Rowe LW. The prevalence of antibody to the viruses of bovine virus diarrhoea, bovine herpes virus 1, rift valley fever, ephemeral fever and bluetongue and to *Leptospira* sp in free-ranging wildlife in Zimbabwe. Epidemiol Infect. (1998) 121:441–9. doi: 10.1017/S0950268898001289, PMID: 9825798 PMC2809544

[70] Pellissier A Rousselot R. Serological investigation on the incidence of neurotropic viruses in some monkeys in French equatorial Africa. Bull Soc Pathol Exotique Filiales. (1954) 47:228–31. PMID: 13182500

[71] Nielsen SS Alvarez J Bicout DJ Calistri P Depner K Drewe JA. Rift Valley fever: risk of persistence, spread and impact in Mayotte (France). EFSA J Eur Food Saf Authority. (2020) 18:e06093. doi: 10.2903/j.efsa.2020.6093 PMC7448016 32874301

[72] Bird BH Khristova ML Rollin PE Ksiazek TG Nichol ST. Complete genome analysis of 33 ecologically and biologically diverse Rift Valley fever virus strains reveals widespread virus movement and low genetic diversity due to recent common ancestry. J Virol. (2007) 81:2805–16. doi: 10.1128/JVI.02095-06, PMID: 17192303 PMC1865992

[73] Davies FG Clausen B Lund LJ. The pathogenicity of Rift Valley fever virus for the baboon. Trans R Soc Trop Med Hyg. (1972) 66:363–5. doi: 10.1016/0035-9203(72)90253-2, PMID: 4558832

[74] Morrill JC Jennings GB Johnson AJ Cosgriff TM Gibbs PH Peters CJ. Pathogenesis of Rift Valley fever in rhesus monkeys: role of interferon response. Arch Virol. (1990) 110:195–212. doi: 10.1007/BF01311288, PMID: 1690534

[75] House C Alexander KA Kat PW O'Brien SJ Mangiafico J. Serum antibody to Rift Valley fever virus in African carnivores. Ann N Y Acad Sci. (1996) 791:345–9. doi: 10.1111/j.1749-6632.1996.tb53541.x, PMID: 8784515

[76] Wilson WC Kim IJ Trujillo JD Sunwoo SY Noronha LE Urbaniak K. Susceptibility of White-tailed deer to Rift Valley fever virus. Emerg Infect Dis. (2018) 24:1717–9. doi: 10.3201/eid2409.180265, PMID: 30124402 PMC6106403

[77] Lutomiah J Omondi D Masiga D Mutai C Mireji PO Ongus J. Blood meal analysis and virus detection in blood-fed mosquitoes collected during the 2006–2007 Rift Valley fever outbreak in Kenya. Vector Borne Zoonotic Dis. (2014) 14:656–64. doi: 10.1089/vbz.2013.1564 25229704 PMC4171391

[78] Gaudreault NN Indran SV Bryant PK Richt JA Wilson WC. Comparison of Rift Valley fever virus replication in north American livestock and wildlife cell lines. Front Microbiol. (2015) 6:664. doi: 10.3389/fmicb.2015.00664 26175725 PMC4485352

[79] Rissmann M Lenk M Stoek F Szentiks CA Eiden M Groschup MH. Replication of Rift Valley fever virus in amphibian and reptile-derived cell lines. Pathogens. (2021) 10:681. doi: 10.3390/pathogens10060681 34072763 PMC8228813

[80] McMillen CM Arora N Boyles DA Albe JR Kujawa MR Bonadio JF. Rift Valley fever virus induces fetal demise in Sprague-Dawley rats through direct placental infection. Sci Adv. (2018) 4:eaau9812. doi: 10.1126/sciadv.aau9812, PMID: 30525107 PMC6281433

[81] Oreshkova N van Keulen L Kant J Moormann RJ Kortekaas J. A single vaccination with an improved nonspreading Rift Valley fever virus vaccine provides sterile immunity in lambs. PLoS One. (2013) 8:e77461. doi: 10.1371/journal.pone.0077461, PMID: 24167574 PMC3805595

[82] Morrill JC Mebus CA Peters CJ. Safety and efficacy of a mutagen-attenuated Rift Valley fever virus vaccine in cattle. Am J Vet Res. (1997) 58:1104–9. PMID: 9328662

[83] Hunter P Erasmus BJ Vorster JH. Teratogenicity of a mutagenised Rift Valley fever virus (MVP 12) in sheep. Onderstepoort J Vet Res. (2002) 69:95–8. PMID: 12092782

[84] Schön K Lindenwald DL Monteiro JT Glanz J Jung K Becker SC. Vector and host C-type lectin receptor (CLR)-fc fusion proteins as a cross-species comparative approach to screen for CLR-Rift Valley fever virus interactions. Int J Mol Sci. (2022) 23:3243. doi: 10.3390/ijms23063243, PMID: 35328665 PMC8954825

[85] Wright D Allen ER Clark MHA Gitonga JN Karanja HK Hulswit RJG. Naturally acquired Rift Valley fever virus neutralizing antibodies predominantly target the Gn glycoprotein. iScience. (2020) 23:101669. doi: 10.1016/j.isci.2020.101669 33134899 PMC7588868

[86] Hopkins KC Tartell MA Herrmann C Hackett BA Taschuk F Panda D. Virus-induced translational arrest through 4EBP1/2-dependent decay of 5'-TOP mRNAs restricts viral infection. Proc Natl Acad Sci U S A. (2015) 112:E2920–9. doi: 10.1073/pnas.1418805112, PMID: 26038567 PMC4460451

[87] Moy RH Gold B Molleston JM Schad V Yanger K Salzano MV. Antiviral autophagy restricts Rift Valley fever virus infection and is conserved from flies to mammals. Immunity. (2014) 40:51–65. doi: 10.1016/j.immuni.2013.10.020, PMID: 24374193 PMC3951734

[88] Mastrodomenico V LoMascolo NJ Cruz-Pulido YE Cunha CR Mounce BC. Polyamine-linked cholesterol incorporation in Rift Valley fever virus particles promotes infectivity. ACS Infect Dis. (2022) 8:1439–48. doi: 10.1021/acsinfecdis.2c00071, PMID: 35786847 PMC9549488

[89] Schneider EH Fitzgerald AC Ponnapula SS Dopico AM Bukiya AN. Differential distribution of cholesterol pools across arteries under high-cholesterol diet. Biochim Biophys Acta Mol Cell Biol Lipids. (2022) 1867:159235. doi: 10.1016/j.bbalip.2022.159235, PMID: 36113825

[90] Hardcastle AN Osborne JCP Ramshaw RE Hulland EN Morgan JD Miller-Petrie MK. Informing Rift Valley fever preparedness by mapping seasonally varying environmental suitability. Int J Infect Dis. (2020) 99:362–72. doi: 10.1016/j.ijid.2020.07.043, PMID: 32738486 PMC7562817

[91] Turell MJ Wilson WC Bennett KE. Potential for North American mosquitoes (Diptera: Culicidae) to transmit rift valley fever virus. J Med Entomol. (2010) 47:884–9. doi: 10.1093/jmedent/47.5.884, PMID: 20939385

[92] Birnberg L Talavera S Aranda C Núñez AI Napp S Busquets N. Field-captured *Aedes vexans* (Meigen, 1830) is a competent vector for Rift Valley fever phlebovirus in Europe. Parasit Vectors. (2019) 12:484. doi: 10.1186/s13071-019-3728-9, PMID: 31619269 PMC6794816

[93] Turell MJ Dohm DJ Mores CN Terracina L Wallette DL Jr Hribar LJ. Potential for North American mosquitoes to transmit Rift Valley fever virus. J Am Mosq Control Assoc. (2008) 24:502–7. doi: 10.2987/08-5791.1, PMID: 19181056

[94] Turell MJ Britch SC Aldridge RL Xue RD Smith ML Cohnstaedt LW. Potential for Psorophora columbiae and *Psorophora ciliata* mosquitoes (Diptera: Culicidae) to transmit Rift Valley fever virus. J Med Entomol. (2015) 52:1111–6. doi: 10.1093/jme/tjv093, PMID: 26336233

[95] Turell MJ Britch SC Aldridge RL Kline DL Boohene C Linthicum KJ. Potential for mosquitoes (Diptera: Culicidae) from Florida to transmit Rift Valley fever virus. J Med Entomol. (2013) 50:1111–7. doi: 10.1603/ME13049, PMID: 24180117

[96] Snoy PJ. Establishing efficacy of human products using animals: the US food and drug administration's "animal rule". Vet Pathol. (2010) 47:774–8. doi: 10.1177/0300985810372506 20551476

